# A 3D Printed, Bionic Hand Powered by EMG Signals and Controlled by an Online Neural Network

**DOI:** 10.3390/biomimetics8020255

**Published:** 2023-06-14

**Authors:** Karla Avilés-Mendoza, Neil George Gaibor-León, Víctor Asanza, Leandro L. Lorente-Leyva, Diego H. Peluffo-Ordóñez

**Affiliations:** 1 Neuroimaging and Bioengineering Laboratory (LNB), Facultad de Ingeniería en Mecánica y Ciencias de la Producción, Escuela Superior Politécnica del Litoral (ESPOL), Campus Gustavo Galindo km 30.5 Vía Perimetral, Guayaquil 090903, Ecuador; kpaviles@espol.edu.ec (K.A.-M.); ngaibor@espol.edu.ec (N.G.G.-L.); 2 SDAS Research Group, Ben Guerir 43150, Morocco; victor.asanza@sdas-group.com (V.A.); leandro.lorente@sdas-group.com (L.L.L.-L.); 3 Faculty of Law, Administrative and Social Sciences, Universidad UTE, Quito 170147, Ecuador; 4 College of Computing, Mohammed VI Polytechnic University, Ben Guerir 47963, Morocco

**Keywords:** bionic hand, electromyography, artificial intelligence, real-time classification, neural network

## Abstract

About 8% of the Ecuadorian population suffers some type of amputation of upper or lower limbs. Due to the high cost of a prosthesis and the fact that the salary of an average worker in the country reached 248 USD in August 2021, they experience a great labor disadvantage and only 17% of them are employed. Thanks to advances in 3D printing and the accessibility of bioelectric sensors, it is now possible to create economically accessible proposals. This work proposes the design of a hand prosthesis that uses electromyography (EMG) signals and neural networks for real-time control. The integrated system has a mechanical and electronic design, and the latter integrates artificial intelligence for control. To train the algorithm, an experimental methodology was developed to record muscle activity in upper extremities associated with specific tasks, using three EMG surface sensors. These data were used to train a five-layer neural network. the trained model was compressed and exported using TensorflowLite. The prosthesis consisted of a gripper and a pivot base, which were designed in Fusion 360 considering the movement restrictions and the maximum loads. It was actuated in real time thanks to the design of an electronic circuit that used an ESP32 development board, which was responsible for recording, processing and classifying the EMG signals associated with a motor intention, and to actuate the hand prosthesis. As a result of this work, a database with 60 electromyographic activity records from three tasks was released. The classification algorithm was able to detect the three muscle tasks with an accuracy of 78.67% and a response time of 80 ms. Finally, the 3D printed prosthesis was able to support a weight of 500 g with a safety factor equal to 15.

## 1. Introduction

The development of prostheses is necessary to improve the quality of life of people who have lost limbs. These artificial devices play a crucial role in replacing missing parts of the body, allowing for the restoration of functionality, mobility, and autonomy of affected individuals [1,2,3]. Additionally, prosthesis provide long-term health benefits, drive technological advancements, and enable adaptation. Continuous research and development in this field are fundamental to further improving the lives of those who depend on these devices.

The hand is one of the most developed organs of the body. It allows humans to interact with their environment through complex movements due to the considerable number of degrees of freedom in its structure (27 in total: four in each finger, five in the thumb and six in the wrist). In addition, it is a fundamental part of physical and social interactions. The upper extremities depend on the hand, so losing one implies a reduction in autonomy, limitations in the development of work and daily activities, and a drastic change in the lifestyle of people [4].

In Ecuador, more than 8% of the population has some need in their upper and/or lower limbs. The cost of a prosthesis, which is around 8000 USD, is excessively high compared to the income of the average Ecuadorian, which, according to *Encuesta Nacional de Empleo, Desempleo y Subempleo* (Enemdu), reached 248 USD in August 2021 [5]. In addition, of the people with physical disabilities, only 17% are employed and most of them are in vulnerable economic situations. Despite several initiatives dedicated to the design and manufacture of prostheses, the needs of existing patients have not been met. The “Las Manuelas” mission, founded in 2007, acquired machinery in 2012 to produce 300 prostheses per month. Until 2019, the prostheses produced did not exceed 10% of the target.

Considering the aforementioned information, it is vital to develop systems that can adjust and optimize gripping patterns and gripping capacity [1] and hand movements according to the needs and preferences of each individual. This will allow for greater customization and comfort in the use of prostheses. Bionic hands, powered by electromyographic (EMG) signals, can interpret and translate the electrical signals generated by residual muscles into precise commands for finger and hand movement [6]. This provides a higher degree of control and a more natural experience when using the prosthesis.

Furthermore, advances in artificial intelligence (AI) have played a crucial role in the design and creation of bionic hands capable of interpreting EMG signals for precise movement control. These techniques use machine learning algorithms, artificial neural networks (ANNs), and computer vision techniques to process EMG signals and enhance prostheses [3], which play a key role in the design of bionic hands [7]. AI has also improved the bionic hands’ ability to learn and adapt as they interact with the environment. By collecting and analyzing real-time data, bionic hands can adjust their movements and grip strength more precisely and efficiently. These advancements have allowed bionic hands to provide better and more sophisticated functionality.

Several studies have focused on the design and manufacture of hand prosthetics that utilize EMG signals and AI for real-time control. For example, Amsüss et al. [8] propose a pattern recognition system for surface EMG signals to control upper limb prosthetics using a trained ANN. Other researchers [9] present a study based on EMG signals acquired from muscles and motion detection through a Human–Machine Interface, designing an upper limb prosthesis using an AI-based controller. In another work [10], hardware design for hand gesture recognition using EMG is developed and implemented on a Zynq platform, processing the acquired EMG signals with an eight-channel Myo sensor. Furthermore, in [11], various methods are applied to detect and classify muscle activities using sEMG signals, with ANNs showing the highest accuracy in recognizing movements among and within subjects. Additionally, in [12], a woven band sensor is manufactured for myoelectric control of prosthetic hands based on single-channel sEMG signals.

The use of artificial intelligence techniques, especially ANNs, has revolutionized the design of bionic hands by allowing control through EMG signals, providing a higher degree of control, functionality, and adaptability. These advancements have had a significant impact on the quality of life of people with amputations or disabilities in their upper limbs. However, despite the progress and promising applications of artificial intelligence in EMG signal processing and other fields, there are still challenges and considerations that need to be addressed. In this regard, this work proposes the design and manufacture of an accessible hand prosthesis powered by EMG signals and controlled by an online neural network. The system demonstrates accuracy in classification and load capacity.

The rest of this manuscript is structured as follows: Section 2 presents some notable related works and provides general information on the types of prosthesis, techniques, and EMG signals and their applications using artificial intelligence. Section 3 describes the system design, the mechanical and control circuit design, data acquisition and analysis, and system integration for controlling a bionic hand. Section 4 gathers the experimental results and presents the discussion. Finally, Section 5 presents the conclusions of this study and states the future work.

## 2. Background and Related Works

This section provides a background and a review of the works related to the main topics addressed in the research.

### 2.1. Types of Prostheses

Upper limb prostheses can be classified into two types according to their functionality.

#### 2.1.1. Passive Prostheses

**Figure 1:**
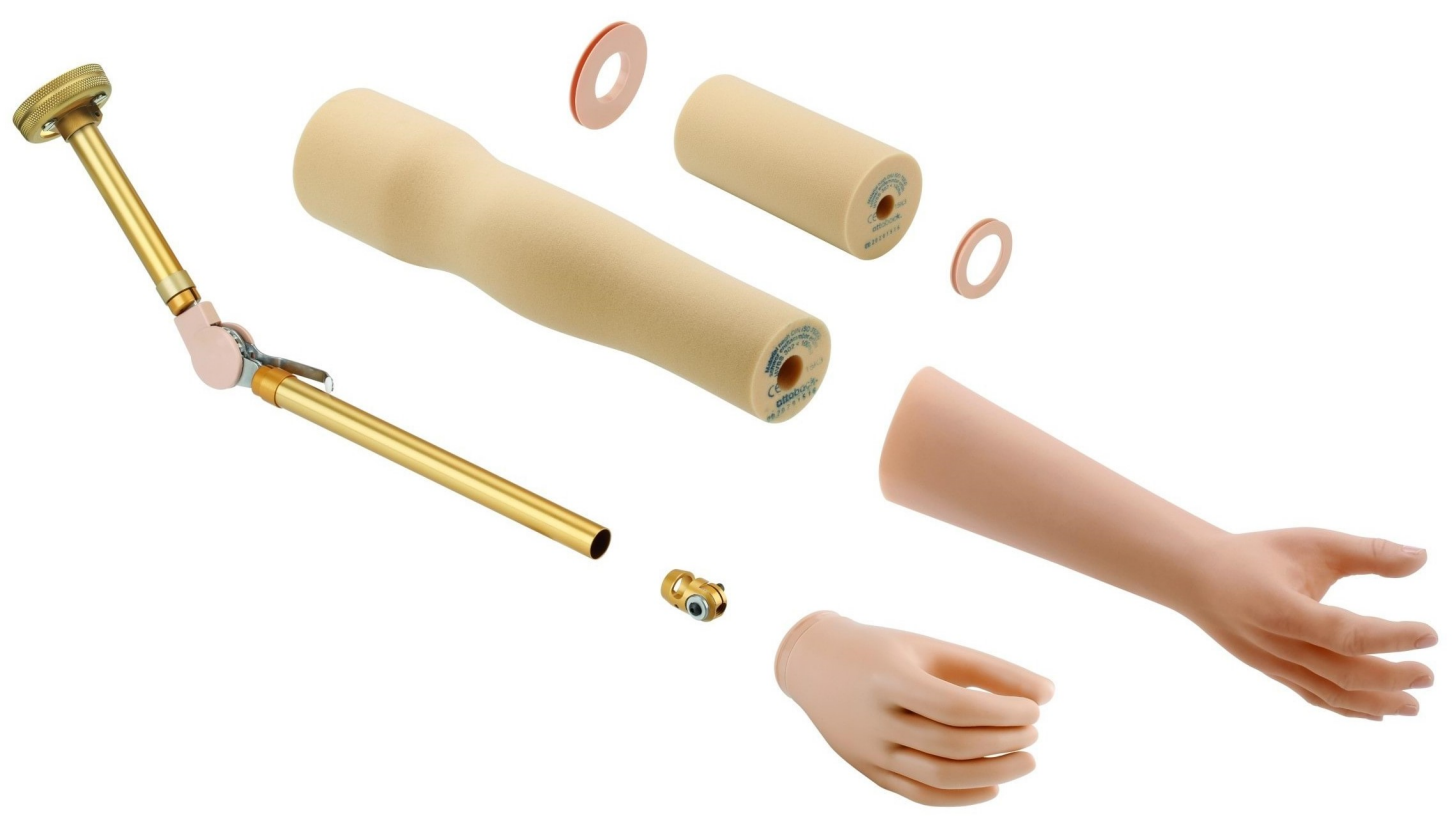
Passive prosthesis [14].

Passive prostheses can be classified into cosmetic or functional prostheses. The cosmetic ones, as seen in Figure 1, are intended to be an aesthetic replacement, simulating the patient’s missing limb section as best as possible [13]; while the functional ones, are intended to assist the subject in very specific activities, which limits the user’s capabilities.

#### 2.1.2. Active Prostheses

**Figure 2:**
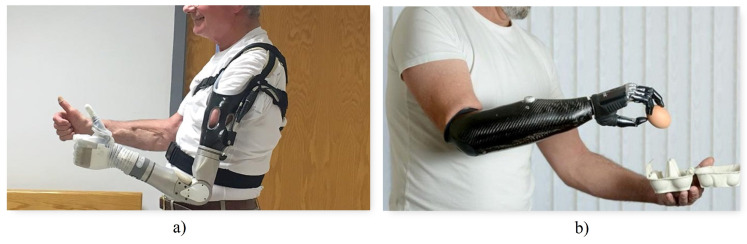
Types of prostheses: (**a**) Active mechanical prosthesis [16] (**b**) Electronic active prosthesis [17].

Active prostheses, as seen in Figure 2a, have mobile joints and can be activated by two types of systems: mechanical actuation and external power supply [4]. Mechanical actuation involves the use of cables and harnesses connected to the available upper limb parts as well as other torso muscles to open, close, or move the prosthesis. The functionality achieved with this method is limited to simple grips and support, with the disadvantage that it requires a considerable amount of effort from the user, fatiguing him or her. On the other hand, externally powered prostheses use batteries to obtain the energy required for movement, as shown in Figure 2b. Additionally, in this type of prosthesis, both the mechanisms that make up the hand and the motion control system are considerably more complex, involving microprocessors and sensors that detect electromyographic signals from the user [15].

### 2.2. Electromyography

Electromyography or EMG is the discipline that deals with the collection and analysis of electrical signals present during muscle contraction [18]. Ideally, the movement of a joint is by voluntary control of the individual and is achieved by motor neurons. This begins in the upper motor neurons found in the cerebral cortex [19]. These send an ionic flux to the lower motor neurons, which start in the spinal cord [19] and are responsible for sending the signal across the membranes of the muscle fibers in question [18]. This generated current is usually in the range of 0 to 10 mV [20].

EMG signals can be used to detect a range of neuromotor diseases, such as muscle atrophy or weakness, chronic denervation, muscle twitching, among others. In turn, they can be used for the voluntary control of robots, computers, machines, and prostheses [21]. In addition, the amplitude of the sEMG signal can be used to examine the timing of muscle activity and the relative intensity or interaction between actively engaged muscles simultaneously [22].

#### 2.2.1. Noise in EMG Signals

EMG signals present certain vulnerabilities, as they travel through different tissues and are affected by various types of noise.

- Inherent noise: Measuring instruments, being electronic equipment, can introduce noise due to their very nature [18]. This can be eliminated with high quality instruments [20].
- Environmental noise: Any electromagnetic device can generate signal noise. In turn, the human body itself contains electromagnetic radiation.
- Movement: When there is any kind of movement, the electrodes—both their pads (which make contact with the skin) and their wires—may add noise to the signals.
- Inherent signal instability: The anatomical and physiological properties of muscles are a type of noise that is impossible to avoid [18]. For this reason, it is important to clean up the noise at a processing stage [20].

#### 2.2.2. EMG Techniques

EMG signals are recorded by means of electrodes, which record their speed and amplitude. For this purpose, there are two types of techniques [23,24].

**Figure 3:**
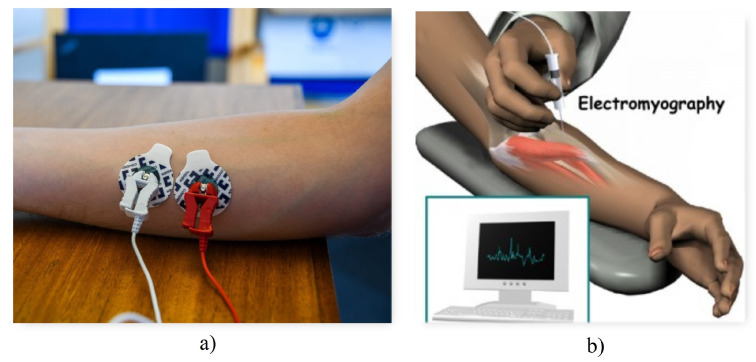
EMG techniques (**a**) Surface EMG technique [25] (**b**) Invasive EMG technique [26].

**Invasive techniques**, as shown in Figure 3b, require the introduction of electrodes to make contact with the inner musculature in order to record minimal current flows.

*Advantages:* By having electrodes that make direct contact with the muscle membrane, more precise signals are obtained.

*Disadvantages:* The complexity of the procedure, the discomfort it implies for the user, the requirement of a professional to carry out the process, and the care required to perform any movement. Therefore, oriented to a daily life application, several factors prevent a comfortable integration between the individual and the prosthesis.

On the other hand, **non-invasive or surface techniques**, as shown in Figure 3a, use surface electrodes placed on the skin.

*Advantages:* It provides much more comfort and freedom to the user, since it does not represent any significant risk in case any pad becomes detached or any electrode becomes disconnected.

*Disadvantages:* They are vulnerable to more sources of noise, such as skin impurities. At the same time, the electrode pads can easily wear out within a few hours.

#### 2.2.3. Sampling Frequency

These signals contain relevant information in the 50–150 Hz range. However, some research corroborates the use of higher sampling frequencies to obtain better results [27]. Using a high-pass filter with a cutoff frequency of 120 Hz allows for the elimination of electrical line noise, among others [28].

### 2.3. Anatomy of the Hand

The human hand is made up of four fingers and a thumb. Each finger is composed of four bones. The three segments that protrude from the palm are called phalanges: distal, middle and proximal from the tip to the base of the finger respectively. The fourth bone of each finger is called the metacarpus, these connect each group of phalanges to a group of bones called carpals located at the base of the palm. The carpals allow the wrist to rotate and move using the radius and ulna as a pivot [29]. This structure gives the hand 27 degrees of freedom: four in each finger, five in the thumb, and six in the wrist. Generally, prosthesis manufacturers limit movement to considerably fewer degrees of freedom due to power, space, weight, and control considerations.

### 2.4. Grip Types

Gripping an object involves grasping or holding it between two surfaces of the hand. While the ways in which objects of different shapes and sizes can be grasped are extremely diverse, there is a general system by which grasping can be classified according to the muscular function required to perform and maintain them [30].

Under the previously mentioned system, the grip can be classified as either a power grip or a precision grip. The power grip usually results in flexion of all finger joints. It may include the thumb to stabilize the object to be grasped, which is held between the fingers and the palm. On the other hand, the precision grip positions an object between one or more fingers and the thumb without involving the palm.

### 2.5. Artificial Intelligence

Artificial intelligence (AI) is the scientific field dedicated to developing intelligent systems that can operate autonomously with minimal human intervention [31]. With the advancements in EMG signal recording techniques and the rapid increase in computational power worldwide, the utilization of AI in processing biological signals is experiencing significant growth [32,33]. Moreover, significant progress has been made in the development of applications for engineering, technology, and medicine, mainly aimed at creating novel solutions that enable innovation and early detection of diseases [34,35,36,37]. Additionally, AI algorithms have been successfully employed in hand gesture recognition [38,39] and pattern recognition in EMG signals for the control of robotic prostheses [40].

While artificial intelligence offers significant potential in EMG signal processing and various domains, addressing challenges related to dataset availability, interpretability, and ethical considerations remains crucial for the responsible and effective use of AI in these applications. It is important to note that in critical applications such as medical diagnostics, transparent and interpretable AI models are essential to build confidence and ensure the reliability of the results.

Within the concept of AI, there is Machine Learning (ML) and, within this, there is Deep Learning (DL).

#### 2.5.1. Machine Learning

Machine learning is based on using algorithms based on mathematics and statistics that are capable of finding patterns in a given set of data [31,41].

There are three types of ML: the first is supervised learning, which requires the use of labeled data in order to extract patterns and associate them to their respective label. Classification and regression are two types of supervised learning, in which the discrete and continuous variables are predicted, respectively.

Unsupervised learning: It does not require labeled data, but rather consists of restructuring the input data and grouping them into subsets of data containing similar characteristics. Clustering and association are two types of unsupervised learning.

Reinforcement learning consists of rewarding or punishing an agent for performing on a certain way within an environment. The agent explores the environment, interacts with it, and learns from the feedback provided.

#### 2.5.2. Deep Learning

Deep learning is based on artificial neural networks, which try to simulate the biological behavior of the human brain. They learn from large amounts of data, which are processed by a set of neurons, to finally yield predictions [31].

#### 2.5.3. Tensorflow Lite

There are several libraries designed for the development of AI models, such as Pytorch, Keras, Tensorflow, etc. However, it was necessary to select a library that would allow the model to be easily deployed on the selected microcontroller (see Section 3.2.3). For this reason, Tensorflow Lite was considered as the best option, since it is specifically designed to deploy pre-trained neural networks on microcontrollers [42]. It has a set of tools developed for the deployment of machine learning models in embedded systems. Among the advantages [43] are

- Latency: no need to access a server; the inference is made on device.
- Connectivity: no internet connection is needed, so it can be used in remote sites.
- Privacy: there is no data exchange, preventing the system from being prone to attacks.
- Size: the models are compressed and their size is reduced.

### 2.6. Design Specifications

The average weight of a hand is 400 g or 0.6 percent of total body weight for men and 0.5 percent for women. However, existing prostheses of similar weight are considered too heavy by users. In a comparative study of myoelectric prostheses, a range of 350 g to 615 g was observed for commercial prostheses and 350 g to 2200 g for research prototypes. However, this range does not represent consistent comparisons because in some cases researchers include the actuation and control system in the total weight, while others consider only the weight of the structure that makes up the hand. While there is no maximum weight specification for prostheses, the consensus is that weight should be minimized, with some groups of researchers defining a limit of up to 500 g [44].

The opening distance of commercial prostheses ranges from 35 to 102 degrees with an average closing speed of 78.2 degrees/s. The average grip force is 7.97 N with a maximum of 16.1 N and a minimum of 3 N. Additionally, the flexibility of the finger mechanism in the bending direction is an important factor in avoiding breakage [44].

### 2.7. Mechanisms

The main mechanisms are separated into central and individual actuation systems. Central systems actuate all five fingers simultaneously with a single actuator, while individual systems dedicate one actuator to each finger and, in some cases, two actuators for thumb rotation and flexion–extension.

**Figure 4:**
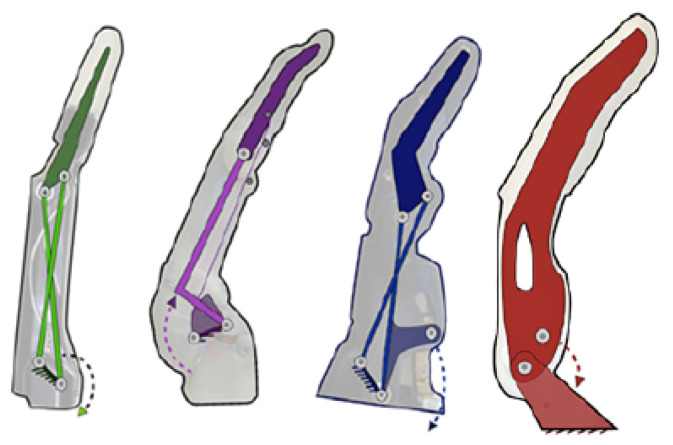
From left to right: mechanisms of the Vincent, iLimb, Bebionic and Michelangelo prostheses [44].

**Figure 5:**
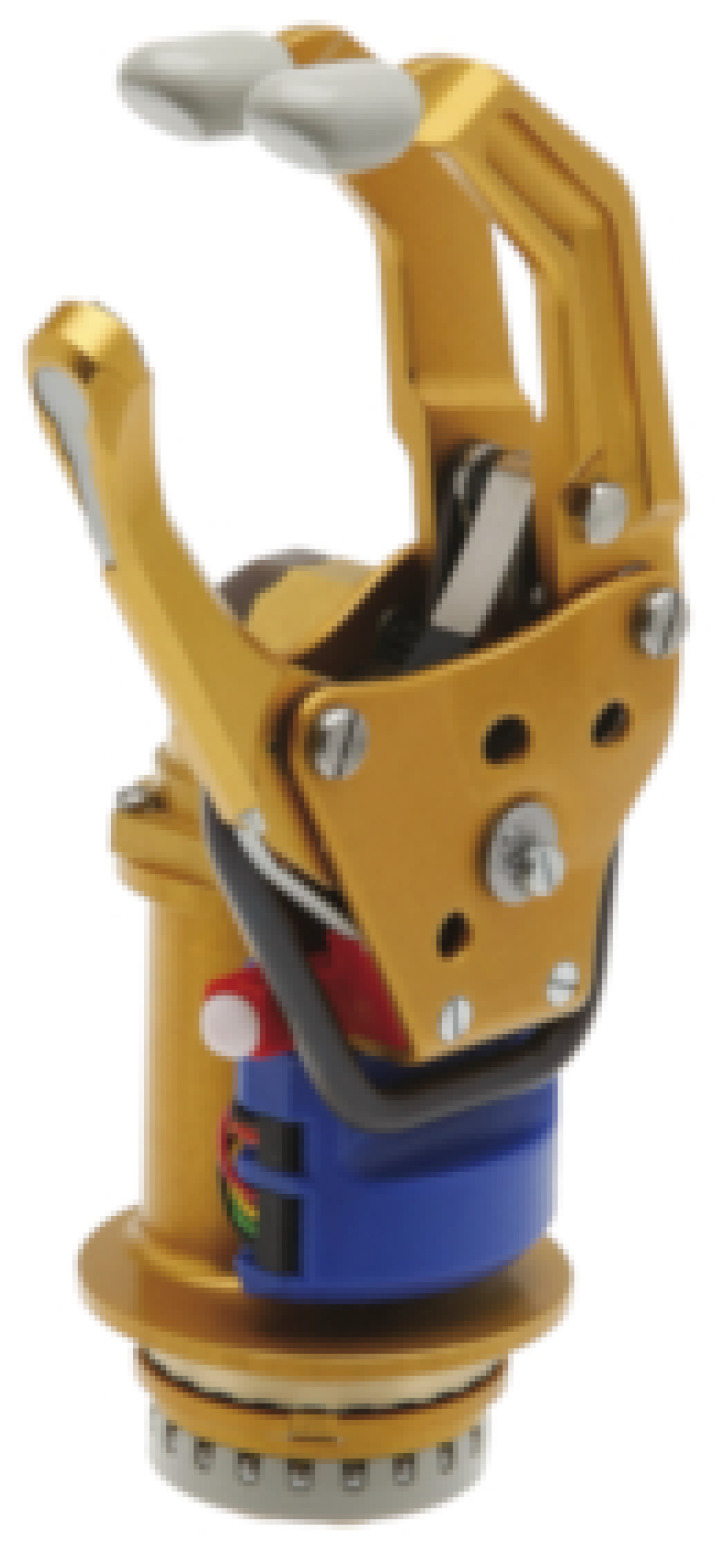
Ottobock’s sensorhand speed [45].

Fingers usually include a proximal joint similar to the metacarpophalangeal joint and a distal joint that encompasses the function of the distal interphalangeal and proximal interphalangeal joints. This type of mechanism can be seen in the Vincent, iLimb, and Bebionic prostheses in the Figure 4. Other variants consist of a single segment as a finger with a metacarpophalangeal joint as can be seen in the Michelangelo prosthesis in Figure 4 and in the SensorHand Speed prosthesis shown in Figure 5.

**Figure 6:**
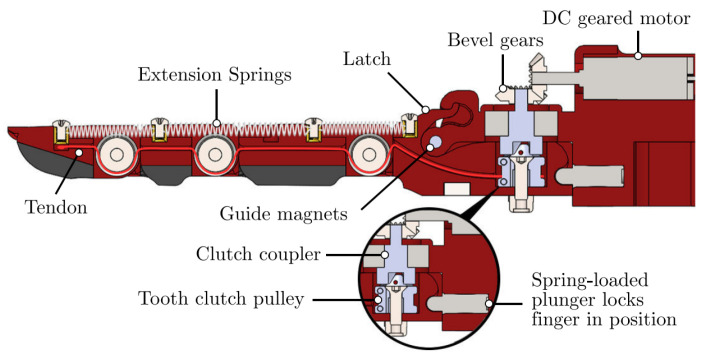
OLYMPIC research prototype [46].

Regardless of the number of joints, the links forming the fingers have a fixed motion relative to each other rather than each joint acting independently. This allows flexion–extension to be achieved by employing various four-bar mechanisms as can be seen in Figure 4. Alternatives for flexion–extension include: ropes as tendons; a set of fingers connected by a link to form a gripper actuated by a single motor as shown in the Figure 5; and a combination of ropes for flexion and springs for extension as seen in the Figure 6.

### 2.8. Sensors

Pancholi and Agarwal [47] developed a low-cost EMG system for arm activity recognition (AAR) acquisition. They found that about 80% of EMG signals were captured efficiently and the overall accuracy for AAR was about 79%. EMG data can be collected from various upper extremity actions, namely HO (open hand), HC (closed hand), WE (wrist extension), WF (wrist flexion), SG (soft grip), MG (medium grip), and HG (hard grip).

### 2.9. Preprocessing

It is important to note that despite the challenges and ongoing research in the field of preprocessing EMG signals, significant progress has been made. Researchers have been able to develop techniques and methodologies to mitigate the effects of noise and improve the quality of acquired signals. Reaz et al. [20] not only identified obstacles in EMG signal acquisition but also proposed methods for detecting and classifying these signals. Their work provides valuable insights into the preprocessing techniques that can be employed to enhance the reliability and accuracy of EMG data analysis. Similarly, other authors [48] conducted a comparative analysis of different configurations for acquiring hand motion EMG signals, achieving a considerable acquisition efficiency of 54%. This demonstrates the effectiveness of their preprocessing approaches in optimizing the signal acquisition process. Furthermore, Fang et al. [49] highlighted the challenges associated with pattern recognition and classification of EMG signals, including issues related to data quality and interpretation. Their research sheds light on the complexities involved in preprocessing EMG data and emphasizes the need for further advancements in this area. In summary, while there are challenges to be addressed in preprocessing EMG signals, researchers have made significant strides in developing techniques and methodologies to overcome these obstacles. Continued research and development in this field are crucial for advancing the accuracy and reliability of EMG signal processing and analysis.

### 2.10. Feature Extraction and Classification

It is worth noting that the selection and utilization of feature extraction and classification techniques for EMG signals depend on various factors, including the specific application, the complexity of the task, and the available computational resources.

While artificial neural networks have shown promising results in EMG signal classification [21,50], other machine learning algorithms, such as Support Vector Machine [51,52], K Nearest Neighbors [53,54], and Multilayer Perceptron [55], have also been successfully employed in this domain [56]. The choice of algorithm often depends on the specific requirements and characteristics of the classification task.

In addition, the emergence of deep learning approaches has facilitated more complex and automated analysis of EMG signals [32,57,58]. These deep neural networks can leverage the hierarchical representation learning capabilities to extract discriminative features directly from the raw EMG data, thereby simplifying the preprocessing stage.

However, regardless of the chosen technique, it is crucial to ensure the quality of the EMG signals. This involves employing appropriate signal cleaning and filtering techniques to remove noise and remove any external disturbances that could interfere with accurate classification [20].

The use of artificial neural networks, along with other machine learning algorithms and deep learning approaches, has achieved excellent results and proven to be effective in the classification of EMG signals. The selection of techniques should be adapted to the specific requirements of the task. Additionally, signal quality and preprocessing steps play a vital role in obtaining reliable and accurate results.

### 2.11. Actuators

Due to space, weight, and energy consumption restrictions, motors are limited to small DC models with high gear reductions.

The prosthesis approach determines the number of actuators included in the prosthesis. Highly functional prostheses are designed with only one or two actuators connected to a transmission system that allows them to assume the main grasping positions with considerable force. On the other hand, anatomically correct prostheses have reduced grip force but are able to mimic a greater number of natural gestures and perform tasks that require greater precision [44].

Additionally, the number of actuators considerably influences the weight of the prosthesis. To counteract this, they are commonly placed on the fingers near the metacarpophalangeal joint or in the center of the palm depending on the choice of main mechanism, as close as possible to each other and other components of considerable weight since the weight distribution influences the weight perceived by the user [44].

## 3. System Design

### 3.1. Mechanical Design

The mechanical design steps are detailed below, starting with the design of the prosthetic fingers whose dimensions are used as the basis for establishing other system parameters.

#### 3.1.1. Finger Design

**Figure 7:**
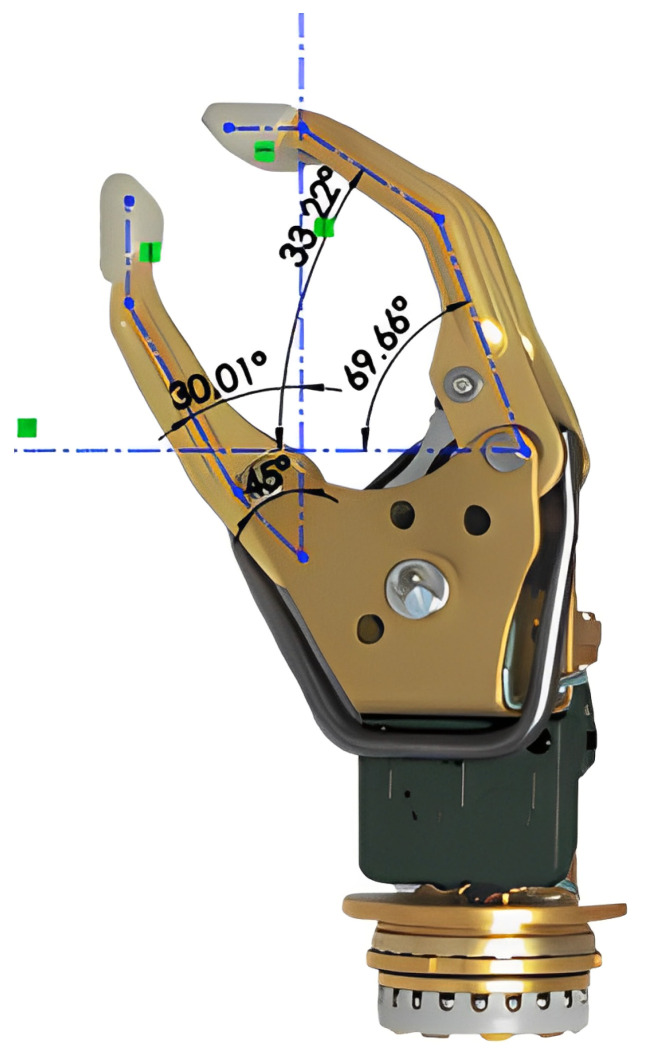
Empirical analysis of interphalangeal angles.

In order to ensure ideal positions for gripping, the angles between the sections representing the distal, medial, and proximal phalanges of the fingers were defined according to Table 1. These are based on the angular difference established for abduction and adduction at the metacarpophalangeal joint [59] and the angle in functional position established for the proximal and distal interphalangeal joint [30]. Additionally, an empirical analysis of the angles between the phalanges observed in the Variplus Speed prosthesis model offered by Ottobock, which resembles the information collected, is presented in Figure 7.

The length of the phalanges was established proportionally to comply with the empirically determined length of the corresponding fingers: 85 mm for the index finger and 60 mm for the thumb. To simplify the design, the index and middle fingers were set to be the same length; only the medial and distal phalanges of the thumb were modeled since they are the ones that protrude from the palm and participate in the grip. Table 2 shows the established dimensions.

#### 3.1.2. Flexion–Extension Mechanism Synthesis

The flexion–extension mechanism was designed using the two-position synthesis method since the abduction and adduction of the fingers can be classified as a coupler output. Based on the desired output, it was known that a four-bar triple rocker would be obtained that could be motor driven by the addition of two intermediate links between the motor coupler and the fingers, resulting on a six-bar Watt’s chain in which the four-bar subchain describes the desired motion [60]. To reduce the unknown variables in the design, lengths for the links that will form part of the fingers were defined from the known length of the selected motor metal coupling, as shown in Table 3.

From these dimensions, the missing links were synthesized graphically using Autodesk Fusion 360. In this program, the separation distance of the links forming part of the index finger and thumb was defined based on the dimensions of the cover designed for the selected engine. Finally, the lengths of the remaining links were adjusted to describe the desired movement and maximum opening, obtaining the lengths shown in Table 4.

**Figure 8:**
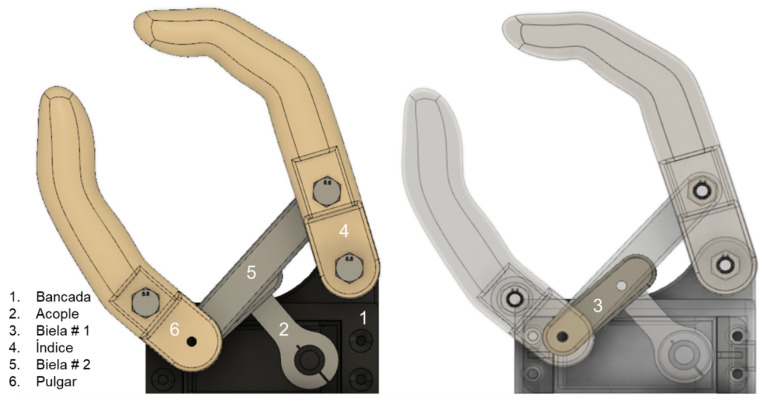
Resulting links.

**Figure 9:**
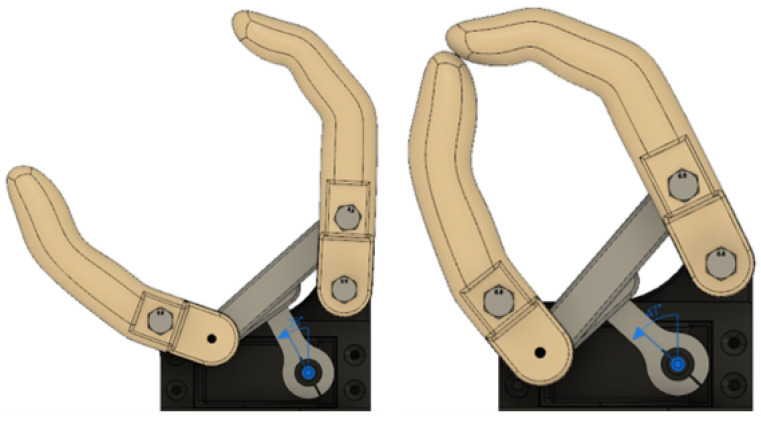
Maximum extension (**left**) and flexion (**right**).

In Figure 8 and Figure 9, the links of the mechanism obtained and their position limits are presented.

#### 3.1.3. Motor Selection

Based on the required torque, the servomotors shown in Table 5 were considered. After analyzing the characteristics of these models and determining that they are in similar ranges in terms of weight, dimensions, and stall current, the DS3218MG model was chosen because the stall torque is double that required for the maximum load, which would provide an additional safety factor to counteract potential losses in the links of the mechanism.

#### 3.1.4. Covers and Turning Base

**Figure 10:**
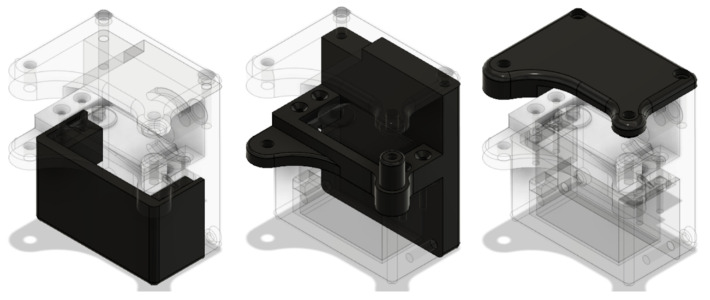
From Left to Right: front cover, rear cover, upper cover.

Based on the dimensions of the selected servomotor, three covers were designed, as shown in the Figure 10, capable of housing it and holding fixed the flexion–extension mechanism to which it would be coupled.

**Figure 11:**
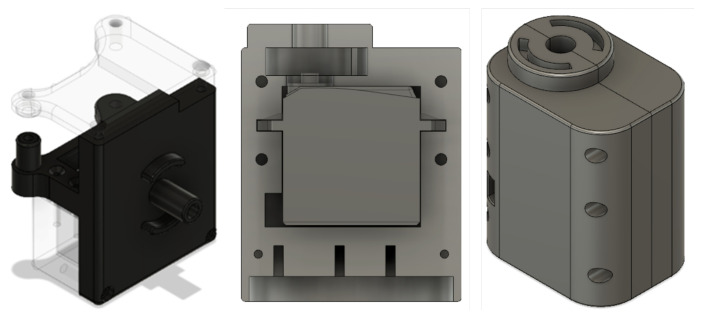
From left to right: Axis, pivot base, perspective pivot base.

Finally, to allow the prosthesis to rotate, a simple shaft connected to the rear cover was designed. This is attached to the rotation base shown in the Figure 11 by means of guide rails and a bearing placed between the top of it and the motor.

### 3.2. Electronic Design

For the design of the control circuit, consecutive stages were established in order to adequately structure both the data recording step and the control of the system in real time.

#### 3.2.1. Sensor

As a first step, a review of the sensors used in similar projects was made. It was found that the MyoWare sensor was the most widely used, due to its accessibility in the market and its low price; its characteristics are shown in Table 6.

#### 3.2.2. Sampling Frequency

Nyquist’s theorem is shown in Equation (1). This states that the sampling frequency of a signal must be at least twice the highest frequency present in the signal. This is the only way to accurately reconstruct the signal.

(1) $$f_{s}\geq 2f_{max}$$

Applying that theorem to electromyography, a sampling frequency of at least 300 Hz should be established, since the relevant information is in the range of 50–150 Hz [27]. However, it has been proven that the application of higher sampling frequencies manages to capture substantial information that allows for improved classification [27]. For this reason, a sampling frequency of 1 kHz was chosen.

#### 3.2.3. Development Board Selection

The following characteristics were considered when choosing the development board.

Communication requirement: Communication between the development board and the computer was crucial, since both times had to be synchronized. For this reason, Bluetooth Low Energy (BLE) was chosen. This communication architecture has been used by several myoelectric signal collection systems [61].

Storage requirement: For the data collection stage, the data had to be stored in the most optimal way without affecting the signal sampling rate. This was high, so the data packets were not small. For this reason, it was decided to store the data locally, using a micro SD card reader module.

The development board must have had *Bluetooth Low Energy* for communication and *GPIO pins*, the connection of sensors and motors, SPI communication for the ESP32 card reader, and I2C communication for a gyroscope. Among the options was one offered by one of the most recognized manufacturers of AIoT (Artificial Intelligence of Things) chips, Espressif. We chose the development board *Esp32 C3 DevKit-M1*, which has the characteristics presented in the Table 7.

#### 3.2.4. Printed Circuit Board

**Figure 12:**
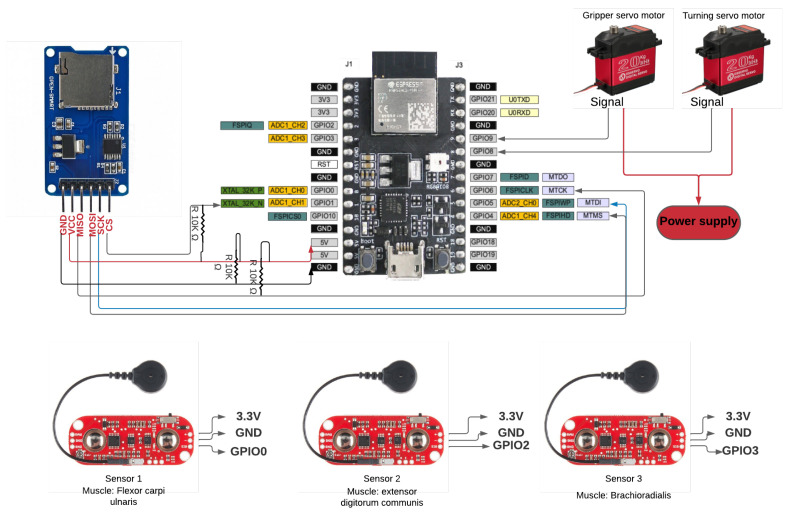
Preliminary electronic circuit design.

Due to the multiple existing connections, it was decided to design a PCB board that would allow a more compact electronic system. The first step was to create a schematic diagram representing all the connections of the electronic elements, as shown in the following Figure 12.

**Figure 13:**
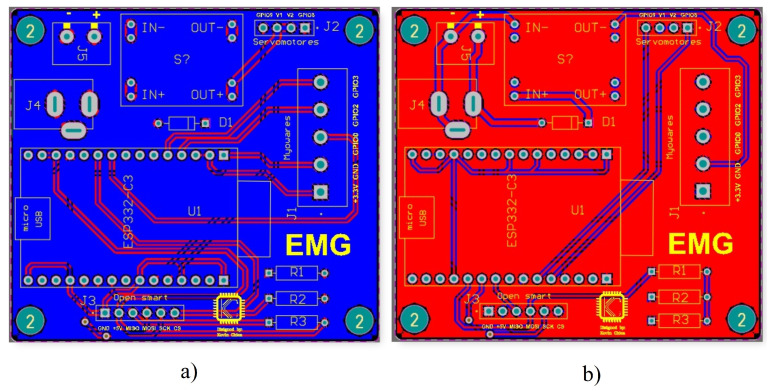
Electronic circuit design. (**a**) Bottom layer (**b**) Top layer.

The board was designed in Altium Designer software, as shown in Figure 13.

**Figure 14:**
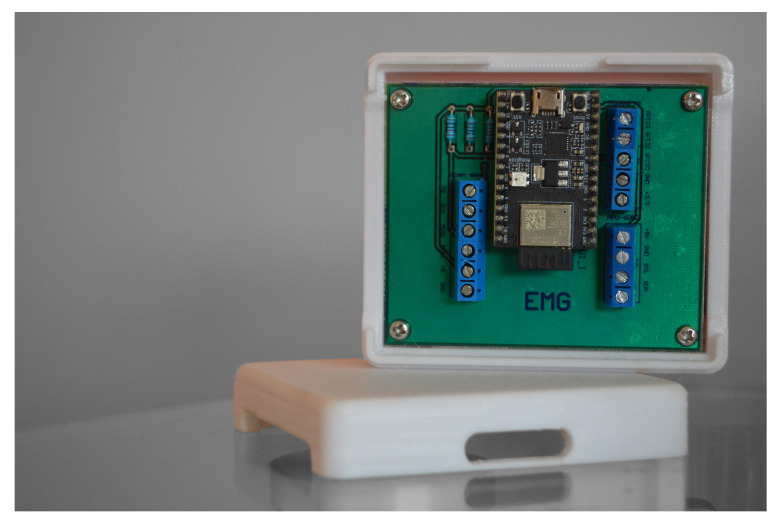
Bakelite printed PCB board.

The electronic circuit was organized on the PCB board; the result is shown on the Figure 14.

Finally, a case was designed to encapsulate the circuit. This allowed decreasing of the noise in the recorded signals and at the same time prevented the circuit from being exposed to factors that could damage it such as shocks, water, external particles, etc.

### 3.3. Data Adcquisition

#### 3.3.1. Experimental Organization

**Figure 15:**
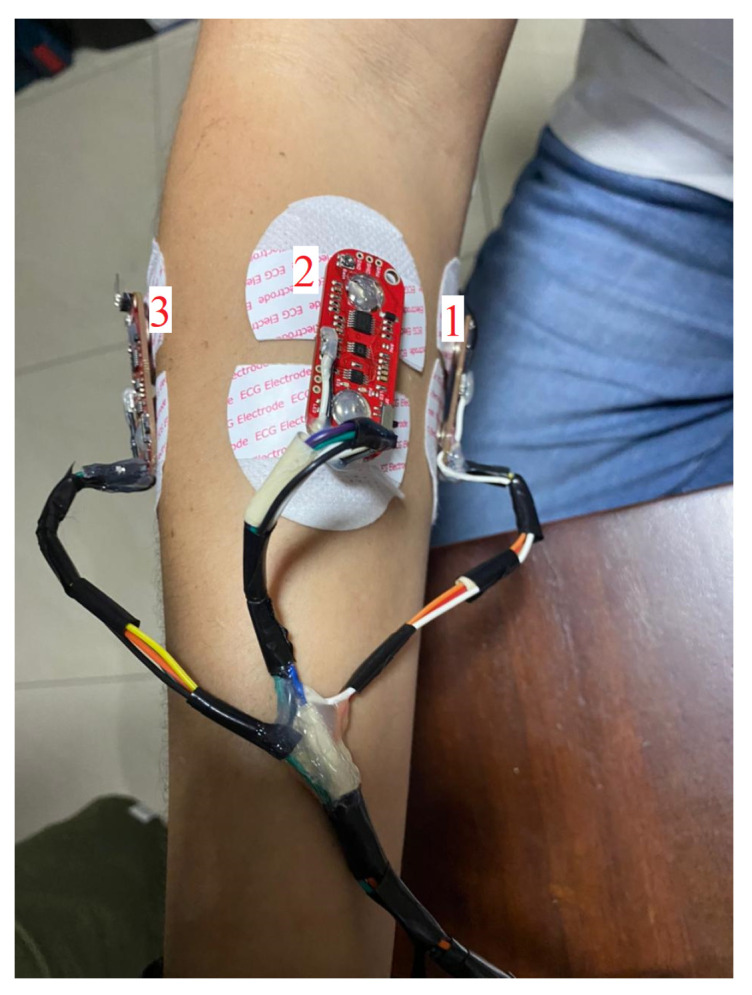
MyoWare sensors positions.

Due to the variability of EMG signals between test subjects (associated with the anatomical and physiological properties of each human being), a single test subject was called for data acquisition. The test subject was received by the personnel in charge of the experiment. This was carried out in a clean, organized space, with natural light and a temperature of around 25 degrees Celsius. We tried to eliminate any type of stimulus that could divert the attention of the test subject, such as intense lights, loud sounds, and people entering/leaving the room. The test subject was asked to sign an informed consent form, detailing that the data recorded would be used strictly for research purposes and would remain anonymous. Then, the procedure of the experiment was explained, as well as how the system worked and that it would not affect the test subject’s health for any reason. The test subject was asked to sit in a reclining chair, which had support for both arms (at 145 degrees) and legs (at 90 degrees). The electrodes were then placed on the patient’s brachioradialis, flexor carpi ulnaris, and common extensor digitorum muscles, as shown in Figure 15. According to the MyoWare sensor manufacturer’s instructions [62], each sensor was placed in the center of the muscle. Finally, the screen showing the experiment was placed at a distance of 20 inches from the test subject.

#### 3.3.2. Experimental Methodology

**Figure 16:**
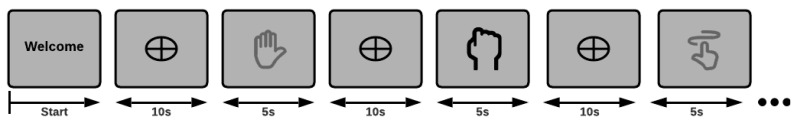
Experimental design.

One run of the experiment lasted 7 min, during which visual signals associated with each of the three muscle tasks were displayed: extension and flexion of the wrist and closing of the hand. Each action appeared 10 times on the screen. Initially, a welcome screen was displayed, in which the test subject was told to start the experiment whenever he was ready by pressing the Space key. Each muscle task lasted 5 s, during which the subject had to contract the muscle and hold it. After the 5 s, 10 rest seconds were given to avoid the risk of muscle fatigue. The process was iterative until the defined time was reached, see Figure 16. The actions were randomly displayed on the screen. Each of the three muscle tasks were recorded at a sampling rate of 1 kHz.

### 3.4. Dataset

The test subject is an Ecuadorian national man who is 31 years old. He has amputation in both upper limbs. The dataset contains 20 files for each muscle task. In total it contains 60 .csv files. Each file has three columns, one for each MyoWare sensor, and another six columns that record the reading of a gyroscope, during the experiment. The dataset can be found at the following link: https://ieee-dataport.org/documents/emg1k-dataset.

All recorded .csv files were stored with class 1, 2 and 3 tags, as detailed below.

- Class 1: wrist flexion
- Class 2: fist
- Class 3: wrist extension

### 3.5. Preprocessing

The data obtained were preprocessed using frequency decreasing techniques, data normalization, and feature extraction. All codes can be found at the following link: The data obtained were preprocessed for use during neural network training, using frequency decreasing techniques, data normalization, and feature extraction. All code can be found at the following link: https://github.com/kaviles22/EMG_SignalClassification.git.

#### 3.5.1. Decrease Frequency

EMG signals are noise sensitive, which is why efforts must be made to smoothen and clean them. For this purpose, the RMS envelope technique was used in Equation (2), which proposes to calculate the quadratic mean in fixed time windows. The objective of this method is to smooth the signal without loosing representative information. This is why the size of the moving window cannot be too large, because a lot of information would be lost, nor too small, because the resulting signal will still present a lot of noise.

(2) $$\sqrt{\frac{\sum_{n=1}^{N}x_{n}^{2}}{N}}$$

**Figure 17:**
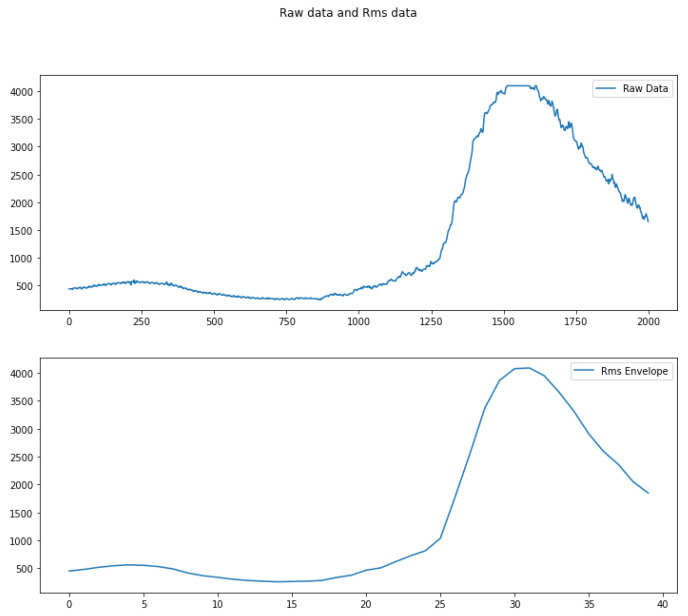
Rms envelope.

Several tests were performed, and it was observed that with very large or very small time window values, the features were not representative; therefore, the time window that yielded the best classification results was chosen, which was 50 ms, as shown in Figure 17.

#### 3.5.2. Normalization

This process was used in order to keep all data within the same range, so that the AI model did not assign a greater importance to some samples rather than others. The peak dynamic method, described in Equation (3), was used, which represents the values of a time window as a division between the value obtained and the maximum value of that time window [63].

(3) $$X_{norm}=\frac{X}{X_{peak}}$$

With this equation, all values remained within the range of [0, 1].

### 3.6. Feature Extraction

At this stage, we kept the features in the time domain, because the established pipeline had to be deployed in real time. Thus, it should not represent a high computational cost for the development board. Two approaches for feature extraction from electromyographic signals were analyzed. Both were tested and the best method was chosen.

#### 3.6.1. Statistical Features

There are different types of features that can be used to represent a biological signal. In this case, six statistical measures were used as features: *Mean Absolute Value, Mean Absolute Value Weight I, Mean Absolute Value Weight II, Median Absolute Value, Variance, and Standard Deviation* [64].

#### 3.6.2. Root Mean Square

Obtain the root mean square in time windows of predefined duration. In this case, a window of 500 ms was chosen from the original window. That is, four RMS values were obtained for each time window (in Section 4.2, it is explained why a final 2-second duration window was chosen).

To evaluate both methods, the classifier (described in more detail in Section 3.7) was used. Its metrics were evaluated using the method *10-fold cross validation*, obtaining the results shown in Table 8.

Based on the results obtained, the rms features were chosen, and it was concluded that the speed of computation of this type of features in real time would represent an advantage over the other method described.

### 3.7. Classification

The capabilities of neural networks have been extensively studied in classification applications using bioelectrical signals, such as visual stimulus detection using EEG-SSVEP signals [65]; detection of imaginative-motor intentions of both hands and both feet, obtaining up to 93.7% of accuracy [66]; analysis of EMG signals to facilitate real-time, off-line monitoring of a prosthetic hand [67]; and EMG signal classification using the Wavelet transform in combination with neural networks, obtaining up to 90.7% accuracy [68].

#### 3.7.1. Model

The following model provides a general representation of the bionic hand control system. This hand is powered by EMG signals and controlled through an online neural network. The model begins by explaining how the EMG signals and hand movements are acquired and their relationship. It then illustrates the signal acquisition process and how they are processed before entering the artificial neural network. Subsequently, the model describes the artificial neural network training process, including adjusting weights and connections to achieve proper control of the bionic hand. In real-time, the artificial neural network receives EMG signals and generates control commands to activate the hand’s motors and actuators, enabling desired movements. Specific implementation details such as activation functions, artificial neural network architecture, and other parameters are discussed in the subsequent subsection (described in more detail in Section 3.7.2). The model’s development involves the following steps:

- Acquisition and preprocessing of EMG signals.
- Feature extraction: relevant features describing muscle activation patterns are extracted from preprocessed EMG signals.
- Training dataset: the dataset consists of pairs of EMG signal features and corresponding movements of the bionic hand. We use 60% of the data for training.
- Artificial neural network structure: input, hidden, and output layers.
- Artificial neural network training: the neural network is fed with the features of EMG signals. It is trained to learn the correspondence between EMG signals and desired movements of the bionic hand.
- Validation and adjustment: after training the neural network, its performance is validated using a test dataset. 20% of the data is allocated for validation, and another 20% is used for testing.
- Implementing the model in the bionic hand.

Below are the inputs and outputs of the model:

Inputs: EMG signals

(4) $$X=[x_{1},x_{2},\ldots ,x_{n}]$$

Outputs: movements of the bionic hand

(5) $$Y=[y_{1},y_{2},\ldots ,y_{n}]$$

Weights and biases:

(6) $$P=\left[P_{1},P_{2},\ldots ,P_{n}\right];b=\left[b_{1},b_{2},\ldots ,b_{n}\right]$$

Hidden layer:

(7) $$S=\sum_{i=1}^{n}X*P_{i}+b_{i}$$

where

- $x_{i}$**:** represents the EMG signal recorded.
- $y_{i}$**:** represents the corresponding movement of the bionic hand.
- $P_{i}$ and $b_{i}$**:** weights and biases of the layers of the neural network.

Feature extraction:

(8) $$F=[f_{1},f_{2},\ldots ,f_{n}]$$

where

- $f_{i}$**:** represents the extracted features from segments of EMG signals.

Training dataset:

(9) $$T=[\left(F_{1},y_{1}\right),\left(F_{2},y_{2}\right)\ldots ,\left(F_{n},y_{n}\right)]$$

where

- $F_{i}$**:** is a feature vector.

Output:

(10) $$Y=\sum_{i=1}^{n}S*P+b$$

where

- ***P*** and ***b*:** weights and biases of the output layer of the artificial neural network.

The mathematical model establishes a relationship between the input EMG signals and the output joint movements of the bionic hand. The artificial neural network is trained to learn this relationship and is used to generate the necessary control signals for the movement of the bionic hand.

#### 3.7.2. Model Training

**Figure 18:**
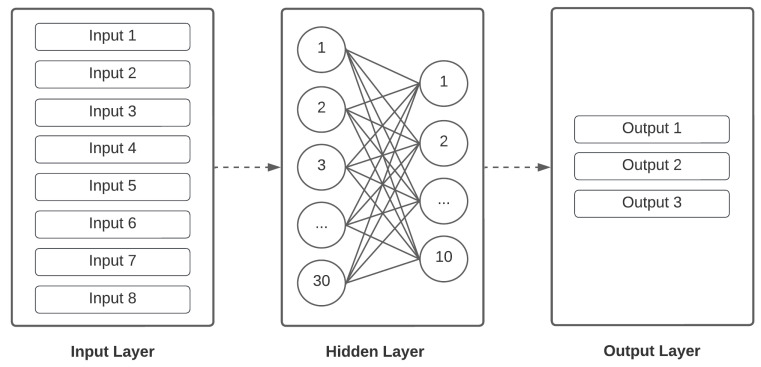
Neural network architecture.

The Tensorflow library, which provides multiple facilities for developing, training, and deploying machine learning models, was used to design the model. Since we needed an online system that would fit on a hardware with limited resources, we decided to go with a simple and small model that could reach high performance and would fit in the microchip. For this reason, a multilayer perceptron of five layers was used; as shown in Figure 18, intermediate dropout layers with a 20% ratio were used to avoid overfitting the model.

The model was trained with 60% of the data, while the remaining 40% was divided equally between test and validation data. The training parameters are presented in Table 9. The final activation function and the loss function were chosen, given that the task was a multiclass classification. The Softmax function yields a probabilistic distribution of N different classes. The loss or cost function, categorical cross-entropy, measures the precision of the results with respect to an expected value for categorical variables. We tested them out, and the results were stable, so we decided to proceed doing a random search to find the optimal hyperparameters. We used a range from 0.01 to 0.0001 for learning rate, epochs were bounded by [100, 200, 300], and the batch size was constant.

After analyzing results, it was decided to train a binary classifier using only tasks 1 and 3, which corresponded to flexion and extension of the wrist, respectively. The training parameters are shown in Table 10.

#### 3.7.3. System Integration

**Figure 19:**
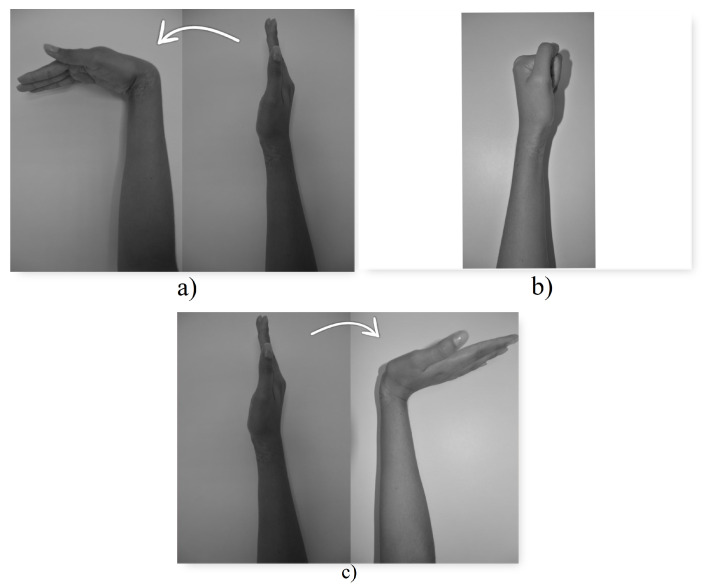
Muscular tasks. (**a**) Class 1 (**b**) Class 2 (**c**) Class 3.

The trained model was integrated into the electromechanical system, allowing the motors to be controlled as follows (see Figure 19):

- When class 1, the wrist flexion task, is detected, the microcontroller moves the axis of the gripper motor 20 degrees clockwise. In this way, the prosthesis gripper closes, simulating the gripping movement of a hand.
- When class 2, the muscle task of closing the hand (making a fist), is detected, the microcontroller moves the axis of the swing motor 30 degrees clockwise. In this way, the base of the prosthesis rotates.
- When class 3 is detected, the wrist extension task, the microcontroller moves the axis of the gripper motor 20 degrees counterclockwise. In this way, the prosthesis gripper opens.

## 4. Results and Discussion

### 4.1. Real Time Classification

The trained model was converted into a C++ source file using the Tensorflow Lite library converter. Finally, a 4756 bytes matrix was obtained. This was stored in the development board in order to be able to carry out the classification.

**Figure 20:**
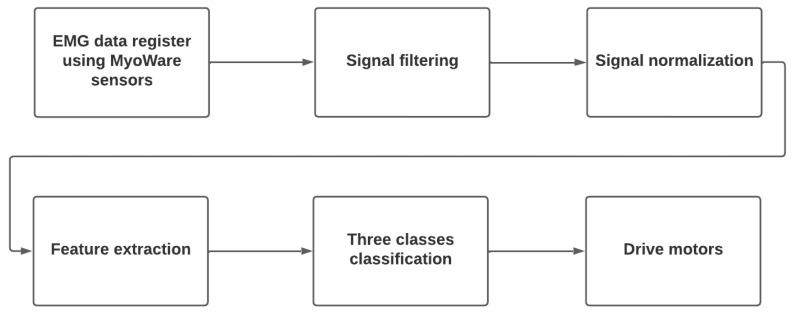
Real time classification flow chart.

Once the model was deployed on the development board, the firmware was capable of sampling, storing, processing, and classifying the data in real time was developed, as shown in Figure 20. The code was written in C++ (https://github.com/kaviles22/EMG_SignalClassification.git).

- EMG data register using MyoWare sensors: data logging using three MyoWare sensors and a microcontroller, at 1 kHz in 2-second windows.
- Signal Filtering: filter the signal noise using the RMS envelope technique.
- Signal Normalization: normalize the three EMG signals to avoid bias during training.
- Feature extraction: extract features (RMS value in this case) in 500 ms windows.
- Three classes classification: make predictions using the compiled and reduced model, previously loaded in the microcontroller.
- Drive Motors: depending on the result of the classification, activate the motors to perform the three actions, depending on the muscle task:
  − Wrist Flexion: closes the prosthesis clamp.
  − Wrist extension: opens the prosthesis clamp.
  − Making a fist: rotates the prosthesis clamp.

### 4.2. Temporal Window

**Figure 21:**
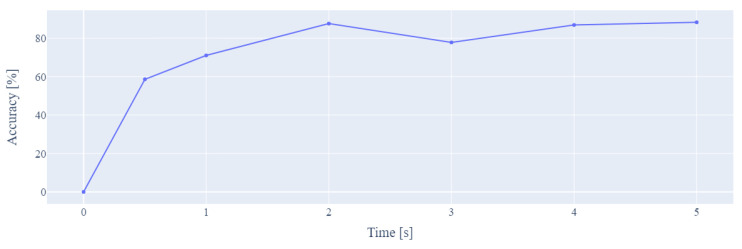
Window size vs. model accuracy.

The system had to have a response time fast enough to be employed in the user’s daily routine. To find this optimal time, all processing steps were run, changing the size of the time window of the EMG signals. Finally, a graph showing the different temporal window sizes vs. the accuracy of the model was obtained, as shown in Figure 21. After analyzing the graph, it was concluded that the most optimal time window duration was 2 s. This complies with a relatively short response time and a relatively high accuracy.

### 4.3. Machine Learning Model Testing

To evaluate the artificial intelligence model, the 10-fold cross validation technique was used. The results are shown in Table 11.

The model classified approximately 78.67% of the classes correctly. When testing the model in real time on the microcontroller, it was preferred not to have many false positives, since in this case, the hand would perform movements without the client’s consent. For these cases in particular, the model had a high performance, since about 80.21% of the positive predictions were correct (precision), meaning that most of the movements performed were voluntary. Approximately 75.67% of the actions were correctly detected (recall).

**Figure 22:**
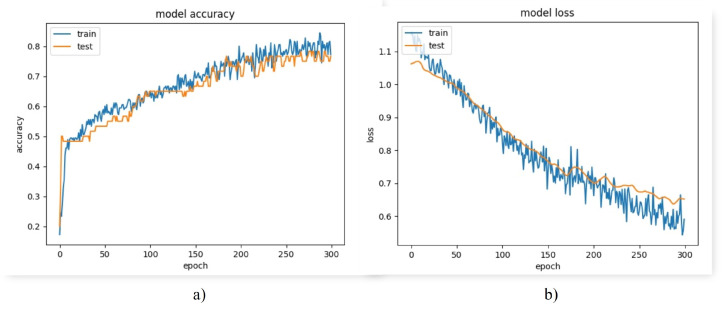
Model performance graphs (**a**) accuracy graph (**b**) loss graph.

Despite having a high accuracy, the loss was high and the model made large errors a few times. As can be seen in Figure 22b, there was both bias and variance in the model. To attack this problem, it was necessary to train the model using more data or to use regularization techniques in the intermediate layers. However, as a first instance, it was decided to analyze the behavior of the model in the different classes.

**Figure 23:**
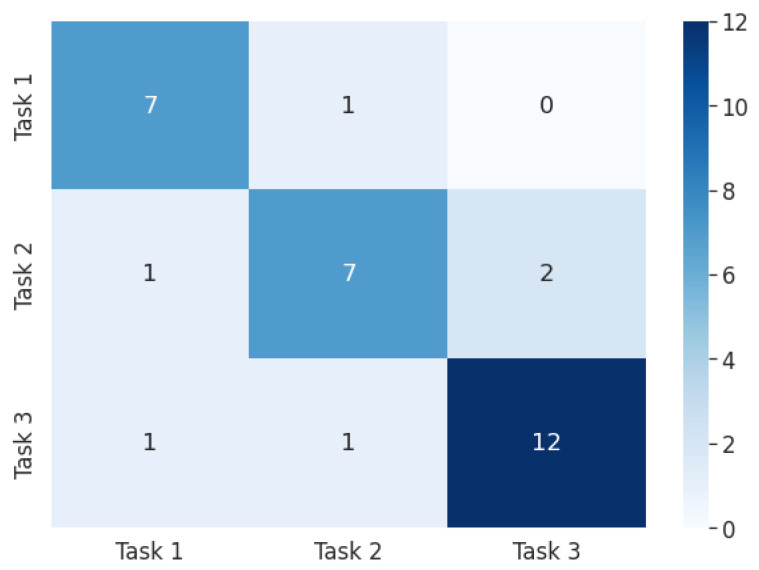
Confusion matrix (multiclass model). Task 1: wrist flexion, Task 2: fist, Task 3: wrist extension.

From the results obtained in Figure 23, it was concluded that the model failed to accurately identify class 2. On the other hand, the accuracies of classes 1 and 3 were relatively high.

**Figure 24:**
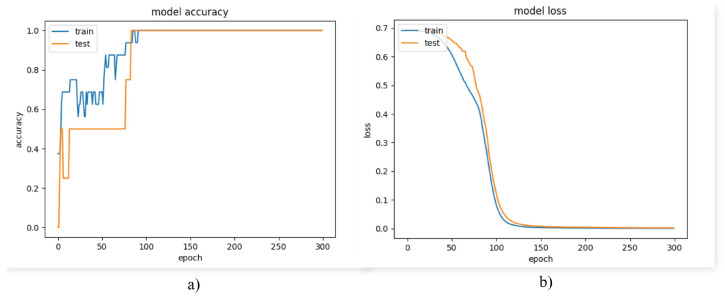
Model performance graphs (binary model). (**a**) Accuracy graph (**b**) Loss graph.

For this reason, it was decided to analyze the behavior of a binary classification model using only classes 1 and 3. Accuracy and loss during training of the binary model are shown in Figure 24a and Figure 24b, respectively. The loss plot shows that the error during training and testing remained close, thus ruling out any possibility of overfitting or underfitting. In turn, this indicates that the bias and variance of the model were low, resulting in a model capable of generalizing effectively. The accuracy reached 100% in less than 300 epochs, while the loss was approximately 0.07. The results are shown in Table 12.

Class 2, associated with the action of making a fist, was not classified efficiently. Unlike the other two tasks, which are opposite actions, flexion and extension, they activate two different muscles. However, class 2 could have been confused with one of the other two muscular actions. Although the action was not the same, it was noticed that the activation of the muscles was very similar.

### 4.4. FirmWare Analysis

The firmware deployed on the ESP32 C3 development board took up 49% of program storage (650 Kb) in memory. The Tensorflow Lite model occupied 4.75 Kb.

### 4.5. Mechanical Analysis

**Figure 25:**
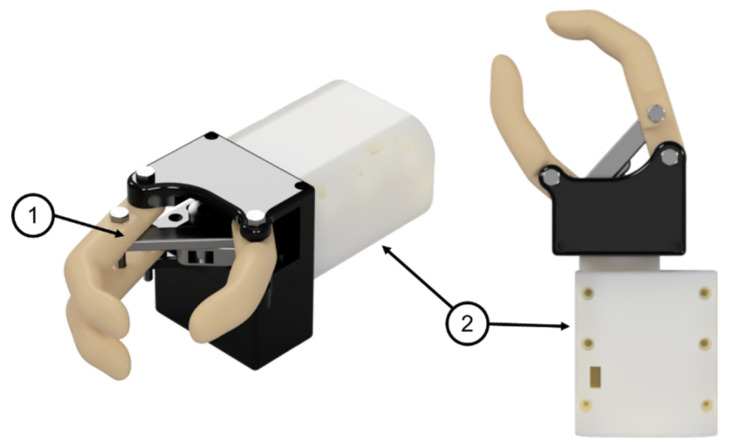
Renderings of the robotic prosthesis: (1) Flexion–extension mechanism (2) Turning base.

The designed prototype is shown in Figure 25. This consists of the flexion–extension mechanism formed by the group of links connected to a servomotor and a turning base that houses another servomotor for rotation. The turning base also functions as a support for the aforementioned assembly.

#### 4.5.1. Dynamic Analysis

**Figure 26:**
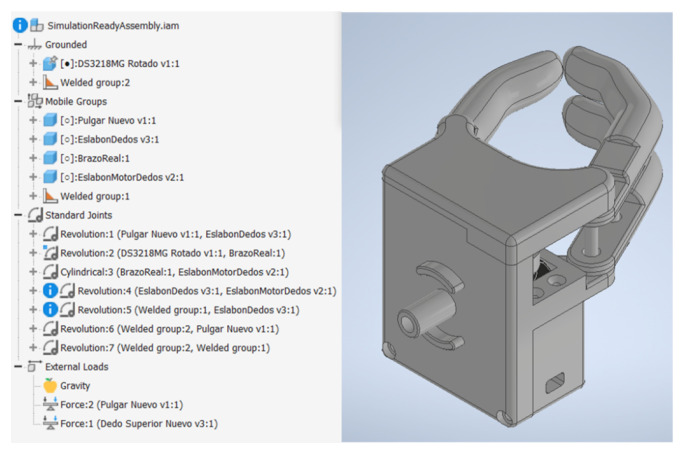
Flexion–extension mechanism in Autodesk Inventor 2022 dynamic simulation environment.

Prior to 3D printing the prototype, a dynamic analysis was performed to observe the behavior of the torque when the prosthesis is subjected to the maximum load during the retraction process. For this, the flexion–extension mechanism was isolated, since this part of the prosthesis is the one that interacts directly with the load. This sub-assembly was imported to Autodesk Inventor 2022 to prepare the joints and forces for the dynamic simulation as can be seen in Figure 26.

**Figure 27:**
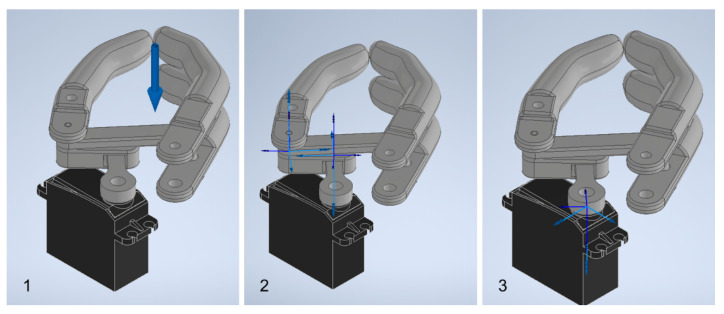
Points of interest: (**1**) Location of loads (**2**) Link joints connected to the motor coupling (**3**) Motor coupling joint. Source: Own elaboration.

Forces 1 and 2 observed in Figure 26 were located at the fingertips, as shown in Figure 27. The joints of interest for the analysis were the coupling joint connected to the motor shaft and the link connected to the coupling directly, defined as Crank #1 in Chapter 2, since these could be used to test whether the required torque would exceed that established during the design stage.

**Figure 28:**
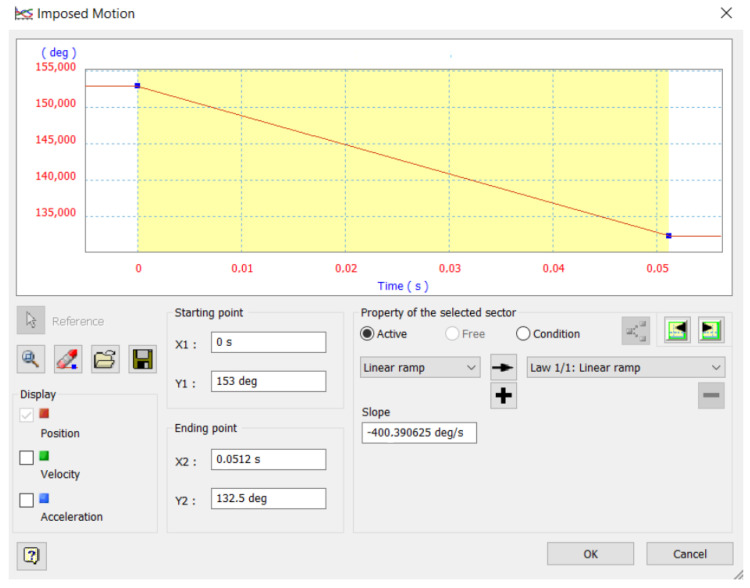
Displacement curve for the imposed movement.

As final step prior to simulation, the displacement curve for the motion that would be imposed on the motor coupling joint was defined. Limits were defined according to the angular positions for the maximum opening and closing, while the slope was approximated to the speed specifications presented in the motor data sheet as shown in Figure 28.

**Figure 29:**
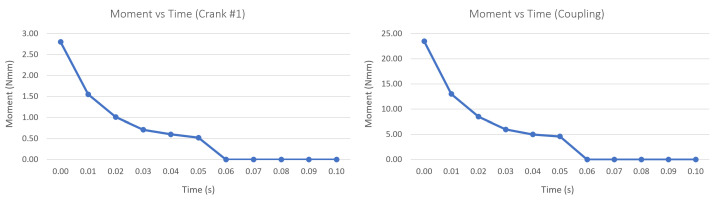
Moment curve for Crank #1 (**left**) and coupling (**right**).

The simulation results are presented in Figure 29. As can be seen, in no joints are the starting moments considerably higher than the required moment; it is assumed that the servomotor will be able to overcome the initial conditions and move the mechanism. Consequently, for the calculation of the safety factor, the starting moment was considered since it represents the maximum moment required to maintain the coupling in equilibrium. This is presented in Table 13 together with the safety factor obtained based on the motor torque (20 kgfcm or 1.961 Nm).

#### 4.5.2. FEA

Once the dynamic analysis was completed, the resulting reactions on the joints of Crank #1 at the beginning of the movement were exported, as shown in the figure, since at this point they reached their maximum value and would allow establishing the safety factor of the link for the worst operating scenario.

**Figure 30:**
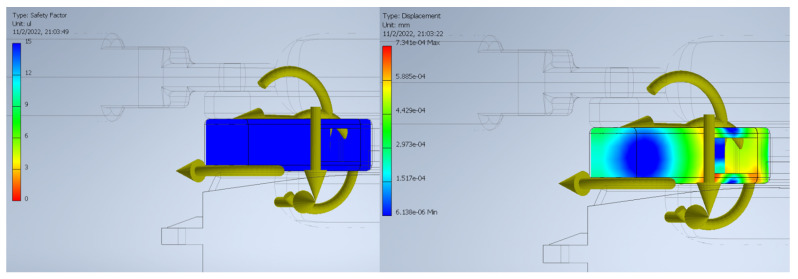
Safety factor (**left**) and link displacement (**right**).

With these reactions, displacements due to the load were observed in the order of 10${}^{-4}$ mm and a safety factor of 15 along the entire body of the link (see Figure 30), so no deformations or fatigue failures are expected due to the maximum load during operation. However, in the event that the hand is subjected to a load above that expected and beyond that allowed by the safety factor, failures would be expected to occur in the lower right side of the link.

#### 4.5.3. Load Tests

**Figure 31:**
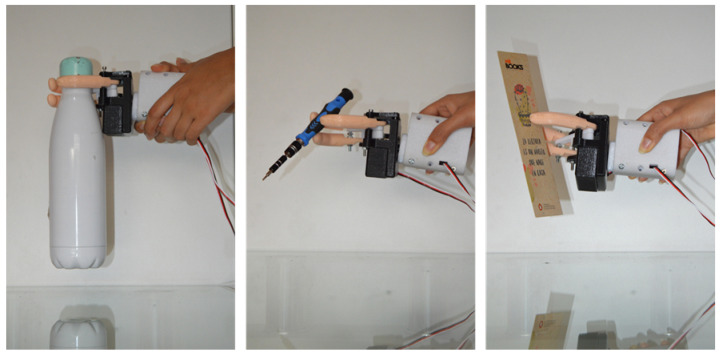
Load tests.

The prosthesis was subjected to different load tests—see Figure 31—to observe the functioning of the mechanism and to check that it was capable of holding the established maximum load of 500 g, as well as holding delicate and small objects.

#### 4.5.4. Prototype Weight

**Figure 32:**
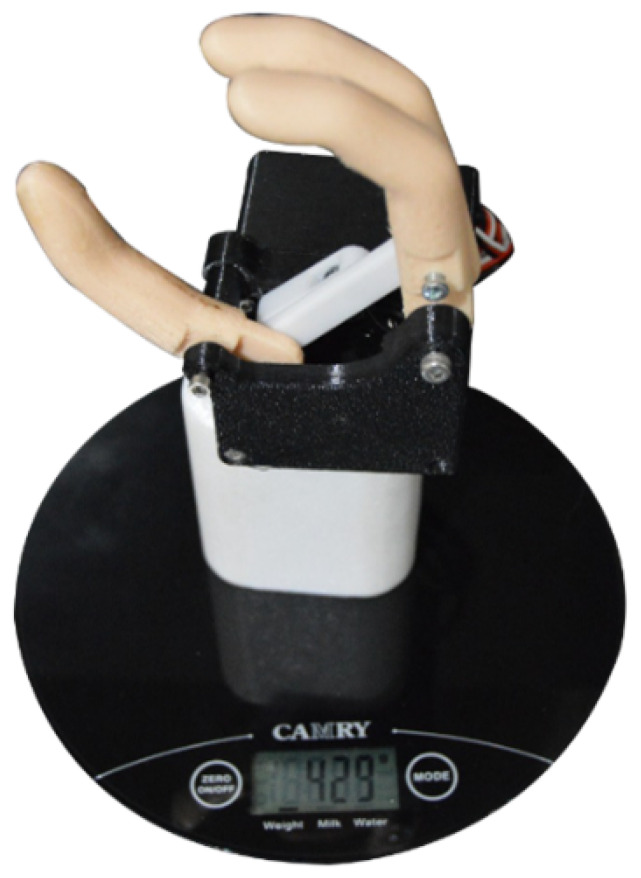
Prototype weight.

To verify that the maximum weight was not exceeded, the printed prototype was placed on a digital scale as shown in the Figure 32, obtaining a total weight of 429 g.

## 5. Conclusions and Future Works

The design of a three-finger prosthesis capable of performing precision and strength grips was presented. This design utilizes a six-bar mechanism to alternate between its position limits and is capable of holding the target load of 500 g, as well as gripping small objects that require greater precision such as pens or screwdrivers. In terms of gripping force, it was estimated that based on the selected motor and the length of the fingers, it is capable of holding the target load of 500 g. It would reach 23 N, which is within the average range of research prototypes.

Additionally, this design was validated by a dynamic simulation of the opening and closing of the gripper and a finite element analysis on the most important link, both carried out using Autodesk Inventor 2022. Based on the resulting starting torque from the dynamic simulation, it was determined that the selected motor was adequate, ensuring that the mechanism will not suffer unforeseen displacements due to the load. On the other hand, the connecting link between the motor coupling and the finger link did not show any significant deformations or fatigue failures, so its design is adequate for the target load. To reduce the weight of the prototype, as well as the cost of printing while maintaining structural integrity, we plan to simplify the covers and turning base in future iterations using the shape optimization tool available in Fusion 360.

The gyroscope, which was used during data collection, did not provide relevant information to the problem. This is due to the fact that during the recording it was ensured that the arm maintained a single position and angle. Therefore, the sensor did not register significant movement changes. Its integration could be useful to explore future topics.

The sampling frequency used in the experiment (1 kHz) stood out compared to commercial EMG signal collection systems, which oscillate at around 128 Hz. This allowed us to work with smaller time windows during preprocessing, to filter the signal without losing representative information, and to characterize the signal efficiently by calculating the RMS values. Additionally, the latter technique allowed us to maintain the temporality of the signals.

Although the overall accuracy of the algorithm was 80.21%, the performance of the model with respect to the action of making a fist was specifically poor compared to the other actions, with an accuracy of 70%. Based on observations, in healthy subjects, this particular action involves both the inner and outer forearm muscle. However, in the test subject, this same action involved only the inner muscle. For example, the wrist flexion and extension actions are opposite actions that activate mostly opposite muscles of the arm: brachioradialis muscle and flexor carpi ulnaris. Therefore, it is easier to discriminate between them. As a solution to this challenge, it is proposed to train the algorithm using more data for it to generalize better across actions.

Since the hardware designed uses the ESP32-C3, its RISC-V based architecture presents advantages in the storage and reading of data in memory, allowing us to obtain a sampling rate of 1kHz for each of the nine variables of interest (three EMG sensors, three acceleration axes, three gyroscope axes). The designed electronic circuit was able to collect data for the training stage and in turn, was used for real-time operation to control the gripper. The ESP32-C3 development board was able to process the EMG signals and drive the gripper servomotors according to the outputs of the AI model.

Unlike EMG systems that may use signal intensity as a trigger, this system is based on an analysis of signal behavior; this provides robustness to the system. If a user presents any symptom of muscle fatigue due to prolonged use, the system would be able to characterize the shape of the signal, regardless of its relative intensity.

Through the analysis of the accuracy in different time windows, it was concluded that using a larger one during data recording does not necessarily represent a significant increase in the accuracy of the model. This is due to the fact that EMG MyoWare sensors have an active filter which prevents the signal from remaining active for a long period of time. Therefore, the most relevant gait is found in the activation of the muscle and not in its deactivation. Thus, reducing the time window could help to reduce the amount of not representative data.

The inference algorithm was able to efficiently classify 95.13% of two of the classes using data collected from one subject. The online classification time of the system was 0.08 s, while a data recording of 2 s of duration was needed, giving a total prosthesis reaction time of 2.18. By using a threshold to consider only predictions with an accuracy greater than 70%, we were able to reduce the number of erroneous predictions between classes. The priority was to reduce false positives as much as possible. In other words, when the subject performed an action, he preferred to keep the gripper still rather than having it move incorrectly. In this case, bias or variance phenomena was not detected in the model as training and testing errors were low.

The project was framed by certain specific objectives and met all the requirements. However, we recognize that the applications are limited to the framework of the project. The hand design could be improved to endure more weight, particularly on the wrist. Regarding the portability, as a future work, it is proposed to design a much more light and compact circuit that can be easily carried on the subject’s arm. Additionally, it is proposed to redesign the connections of the development board in order to reduce the noise generated in the EMG signals, due to physical factors, as much as possible. Regarding the artificial intelligence model used, it is proposed to evaluate the use of deep neural networks capable of generalizing between data from different test subjects, reducing the preprocessing time, and increasing the accuracy of the model. In addition use of recurrent neural networks to explore the capability of expanding the time window analysis can be studied.

For more reality-oriented implementations, it is proposed to analyze the use of the gyroscope. In addition, since the test subject does not remain static, but requires the use of the prosthesis while in motion, it is proposed to use dry electrodes, since adhesive sensors tend to cause skin irritation.

## Figures and Tables

**Table 1:** Interphalangeal angles.

Finger	Proximal Phalanx	Medial Phalanx	Distal Phalanx
Thumb	30°	35°	5°
Index/Middle	70°	30°	5°

**Table 2:** Phalanx length.

Finger	Proximal Phalanx Length [mm]	Medial Phalanx Length [mm]	Distal Phalanx Length [mm]
Thumb	-	35	25
Index/Middle	40	25	20

**Table 3:** Established dimensions for links.

Link	Name	Length [mm]
2	Coupling	35
4	Index	20.5
6	Thumb	16

**Table 4:** Dimensions determined through graphic synthesis.

Link	Name	Length [mm]
3	Crank 1	25
5	Crank 2	52

**Table 5:** Motor characteristics.

Model	MG995R	DS3218MG	DS3225MG
Imagen	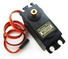	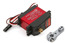	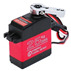
Brand	TowerPro	DSSERVO	DSSERVO
Stall torque [kgf·cm]	9.4–11	18–21	21–24.5
Weight [g]	55	60	60
Operating voltage [V]	4.8–7.2	4.8–6.8	5–6.8
Dimmensions [mm]	40.7 × 19.7 × 42.9	40 × 20 × 40.5	40 × 20 × 40.5
Stall current [A]	1.2	1.8–2.3	1.9–2.3

**Table 6:** Technical characteristics of the MyoWare sensor.

**Supply voltage**	2.9–5.7 [V]
**Power supply current**	9 [mA]
**Various features**	-Raw signal
	-Filtered signal
	-Adjustable gain

**Table 7:** Features of Esp32 DevKit-M1.

**Bluetooth LE**	Yes
**I2C**	Yes
**SPI**	Yes
**GPIO**	22 pins
**ADC**	6 pins
**Price**	$15
**Flash**	4 MB
**Processor architecture**	RISC-V (single core)

**Table 8:** Evaluation of feature extraction methods.

	Rms Features	Custom Features
**Training time [s]**	23.29	22.12
**Accuracy**	78.13%	71.87%
**Recall**	75%	68.75%
**Precision**	80%	76.49%

**Table 9:** Hyperparameters and functions used in model training.

**Activation function**	Softmax
**Loss function**	Categorical cross-entropy
**Optimization algorithm**	Adam
**Learning rate**	0.001
**Epochs**	300
**Batch size**	8

**Table 10:** Hyperparameters and functions used in binary model training.

**Activation function**	Sigmoid
**Loss function**	Categorical cross-entropy
**Optimization algorithm**	Adam
**Learning rate**	0.001
**Epochs**	300
**Batch size**	8

**Table 11:** Model evaluation metrics (multiclass model).

**Accuracy**	78.67%
**Precision**	80.21%
**Recall**	75.67%
**Loss**	0.61
**Training Time**	13.68 [s]
**Inference Time**	0.09 [s]

**Table 12:** Model evaluation metrics (binary model).

**Accuracy**	95.13%
**Precision**	88.37%
**Recall**	99.85%
**Loss**	0.07
**Training Time**	13.12 [s]
**Inference Time**	0.08 [s]

**Table 13:** Dynamic simulation results.

Moment [Nm]	Part	Safety Factor
0.02347	Coupling	83.55

## Data Availability

Data regarding images and annotations can be accessed at: repository name: *ieee-dataport*; data identification number: https://doi.org/10.21227/ppsg-g354; direct URL to data: https://ieee-dataport.org/documents/emg1k-dataset; accessed date: 15 November 2022.
